# Plant nutrient quality impacts survival and reproductive fitness of the dengue vector *Aedes aegypti*

**DOI:** 10.1186/s13071-020-04519-y

**Published:** 2021-01-04

**Authors:** Vincent Odhiambo Nyasembe, David Poumo Tchouassi, Martha Njeri Muturi, Christian W. W. Pirk, Catherine L. Sole, Baldwyn Torto

**Affiliations:** 1 International Centre of Insect Physiology and Ecology (Icipe), Nairobi, Kenya; 2 Department of Bioscience, Kenya Medical Research Institute-Wellcome Trust, Kilifi, Kenya; 3 Department of Zoology and Entomology, University of Pretoria, Hatfield, South Africa

**Keywords:** Plant nectar, Plant sap, Survival, Fecundity, Hatching-rates, Amino acids, Dengue

## Abstract

**Background:**

In a recent study using DNA barcoding, we identified the plants fed upon by four Afro-tropical mosquito species that vector dengue, malaria, and Rift Valley fever. Herein, we have expanded on this study by investigating the role of three of the plants, *Pithecellobium dulce* (Fabaceae), *Leonotis nepetifolia* (Lamiaceae), and *Opuntia ficus*-*indica* (Cactaceae), on the survival, fecundity, and egg viability of the dengue vector *Aedes aegypti*.

**Methods:**

We tested these effects using females that received (i) an initial three rations of blood meals and (ii) no blood meal at all. Two controls were included: age-matched females fed on glucose solution with or without an initial blood meal and those fed exclusively on blood meals. Data were collected daily over a 30-day period. The amino acid contents of *Ae. aegypti* guts and their respective diets were detected by coupled liquid chromatography-mass spectrometry.

**Results:**

Females fed on *P. dulce* and an exclusively blood meal diet had a shorter survival than those fed on glucose. On the other hand, females fed on *L. nepetifolia* survived longer than those fed exclusively on blood meals, whereas those fed on *O. ficus*-*indica* had the shortest survival time. With an initial blood meal, females fed on *L. nepetifolia* laid 1.6-fold more eggs while those fed on the other diets laid fewer eggs compared to those fed exclusively on blood meals. Hatching rates of the eggs laid varied with the diet. Mass spectroscopic analysis of gut contents of mosquitoes exposed to the different diets showed qualitative and quantitative differences in their amino acid levels.

**Conclusion:**

Our findings highlight the central role of plant nutrients in the reproductive fitness of dengue vectors, which may impact their disease transmission potential.

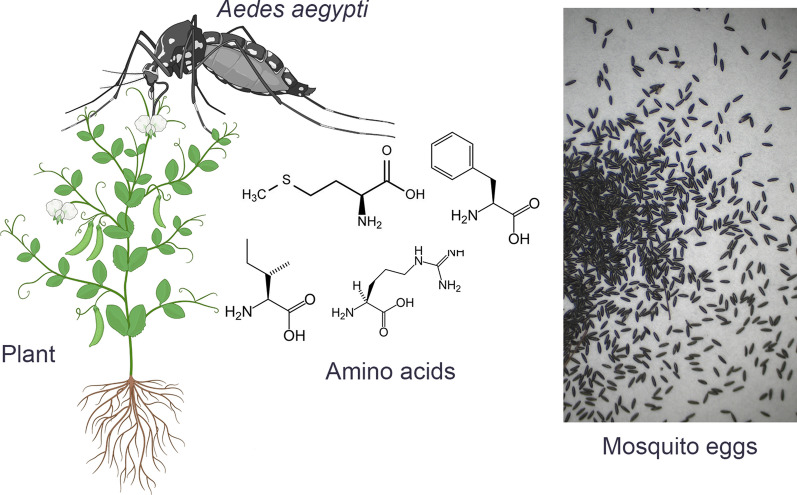

## Background

The last 2 decades have seen the resurgence and spread of arboviruses such as dengue, Zika, and chikungunya viruses, which are vectored by *Aedes* mosquitoes. Although the number of dengue cases are underreported and misclassified [1], the number of global infections is estimated at 390 million annually, with about 50–100 million cases manifesting clinically [2, 3]. The geographic expansion of dengue, which is caused by four dengue virus serotypes (DENV 1-4), has been characterized by increases in case incidence, epidemics, and super-endemicity, with more frequent severe forms of dengue [4, 5]. The recent outbreak of Zika in South and Central America and the Caribbean region attests further to the continued geographic spread of arboviral diseases [6, 7]. Zika was first identified in *Rhesus* monkey in Uganda in 1947, with the first human cases detected in 1952 in Uganda and Tanzania [8]. The Zika outbreaks in Brazil and Colombia in 2015 and its subsequent spread to 13 other countries in the Americas, along with other outbreaks in the Pacific (Yap, 2007; French Polynesia, 2013) and Africa (Cape Verde, 2015), highlight the growing concern about the rapid expansion of arboviral diseases [9]. The overarching commonality among these diseases is that there are currently no specific drugs for their treatment and no viable vaccines available [10]. This makes effective vector control the mainstay for prevention and control of these diseases.

The geographic expansion of these diseases closely follows the tropical and subtropical distribution of their primary vectors, *Aedes aegypti* and *Aedes albopictus*. The spread has been attributed to a range of factors including climate change, uncontrolled urbanization, globalization, travel, trade, socioeconomics, and the ability of these viruses to evolve [5, 11]. In addition, factors contributing to the resilience of *Ae. aegypti* populations such as insecticide resistance, ability of eggs to withstand desiccation, ability of adults to adapt to environmental modifications, and their behavioral plasticity have contributed to the sustenance or even expansion of *Ae. aegypti* populations [12, 13]. Overall, the continued expansion of the geographic range of these mosquito species and the pathogens that they transmit calls for a detailed understanding of vector and disease ecologies in a renewed effort to develop innovative management strategies.

Plant feeding is emerging as a key ecological factor in the biology of several mosquito species including *Aedes* species [14–17]. While plant feeding pre-dates blood feeding in insects, blood sucking arthropods are thought to have adopted the latter trait during evolution to enhance the propagation of their progeny [18]. Among different mosquito species, intermittent plant feeding in females has long been documented but its role with respect to reproductive fitness has been downplayed by different studies [19–22]. Central to this dogma are *Ae. aegypti* and *Anopheles gambiae*, the two highly anthropophilic and most important disease vectors. Variably low fructose levels detected in field-collected females of these species accompanied by their tendency to have multiple blood meals has led to the proposition that they seldom feed on plants but depend on human blood for both their metabolic processes and reproduction. However, recent evidence where more sensitive trapping strategies and analytical approaches were used shows higher plant feeding frequencies in these two species [16, 17, 23].

Several studies have demonstrated the central role played by plant sugars in male and female mosquito survival, mating competence, and flight activity. In addition, there has been substantial effort to identify plant species fed upon by different mosquito species. These efforts have been greatly boosted by the advent of highly sensitive analytical techniques such as plant DNA barcoding and mass spectrometry, which provide secure host plant identification and authenticate their trophic association [24, 25]. Molecular approaches have recently been used to identify plant species fed upon by important disease vectors such as *An. sergentii* [23], *An. gambiae, Ae. mcintoshi, Ae. ochraceus* and *Ae. aegypti* [16], and phlebotomine sand flies [26, 27] in their natural habitats. Evidence of more frequent plant feeding among these vectors and the identification of host plant species further augment the proposition of their central role in vector population dynamics. However, beyond a few studies linking plant feeding to mosquito survival, little is known about the nutritional contribution of plants to vector fitness and population dynamics.

Building on our recent identification of natural host plants of four Afro-tropical mosquito species [16], we sought to elucidate the role of plant nutrition using three of the identified plants on survival and reproductive fitness of *Ae. aegypti*.

## Methods

### Experimental animals

Adult *Ae. aegypti* obtained from eggs collected in Kilifi (3.6333° S, 39.8500° E) in the coastal region of Kenya endemic for dengue (Sang and Dunster, 2001) were used. The eggs were collected by placing black ovicups lined with brown ovistrips in pre-identified *A. aegypti* breeding sites overnight. The collected eggs were either hatched immediately or carefully dried and transported to *icipe* laboratories in Nairobi. The hatching larvae were reared in plastic trays (25 cm long × 20 cm wide × 14 cm high) to adults with a daily ration of Tetramin fish food (Tetramin1, Melle, Germany) of 0.3 g/100 larvae/day. The rearing room was maintained at a temperature of 28 (± 1)  °C and relative humidity of 80 (± 5)  % and a photoperiod of 12: 12 (light: dark) hours. The haplotypes of the emerging adults were all confirmed to be *Ae. aegypti aegypti* (hereafter referred to as *Ae.* aegypti) as they all had white scales on the first abdominal tergite (McClelland,1960). The adults, 1–2 days old with no prior exposure to any other nutrient source, were used in survival and fecundity assays.

### Plant materials

Plant species identified as natural host plants of the four mosquito species from our previous study [16] were used in these assays although specific for the vectors from their respective ecologies. These included *Pithecellobium dulce* (Roxb.) Benth (Fabaceae; *Ae. aegypti* host plant), *Leonotis nepetifolia* (L.) R.Br (Lamiaceae; *An. gambiae* host plant) and *Opuntia ficus*-*indca* (L.) Mill (Cactaceae; *Ae. mcintoshi* and *Ae. ochraceus* host plant).

Studies with *P. dulce* were conducted at the KEMRI-Wellcome Trust laboratories in Kilifi Kenya (3.6333^o^ S and 39.8500^o^ E), where both *Ae. aegypti* and its host plant *P. dulce* co-occur. *P. dulce* is a perennial tree reaching a height of about 10–15 m; hence, we were not able to obtain it as a potted plant. Consequently, fresh cuttings of its leaves, newly blossomed flowers and young pods were used. These were changed daily over the 30-day experimental period.

Studies with *L. nepetifolia* and *O. ficus*-*indica* were conducted at *icipe* in Nairobi. Wild growing *L. nepetifolia* (obtained from Ahero, western Kenya; 0^o^10’S, 34^o^55′E) and *O. ficus*-*indica* (obtained from Ijara, northeastern Kenya; 1.5988^o^ S and 40.5135^o^ E), were transplanted into pots (D 25 × W 27 x H 30 cm) and transported to *icipe* laboratories in Nairobi. They were used when they started to blossom.

All the experiments were conducted under controlled conditions as described above for mosquito rearing.

### Survival, fecundity, and egg hatchability of *Aedes aegypti* on different host plants

**Fig. 1:**
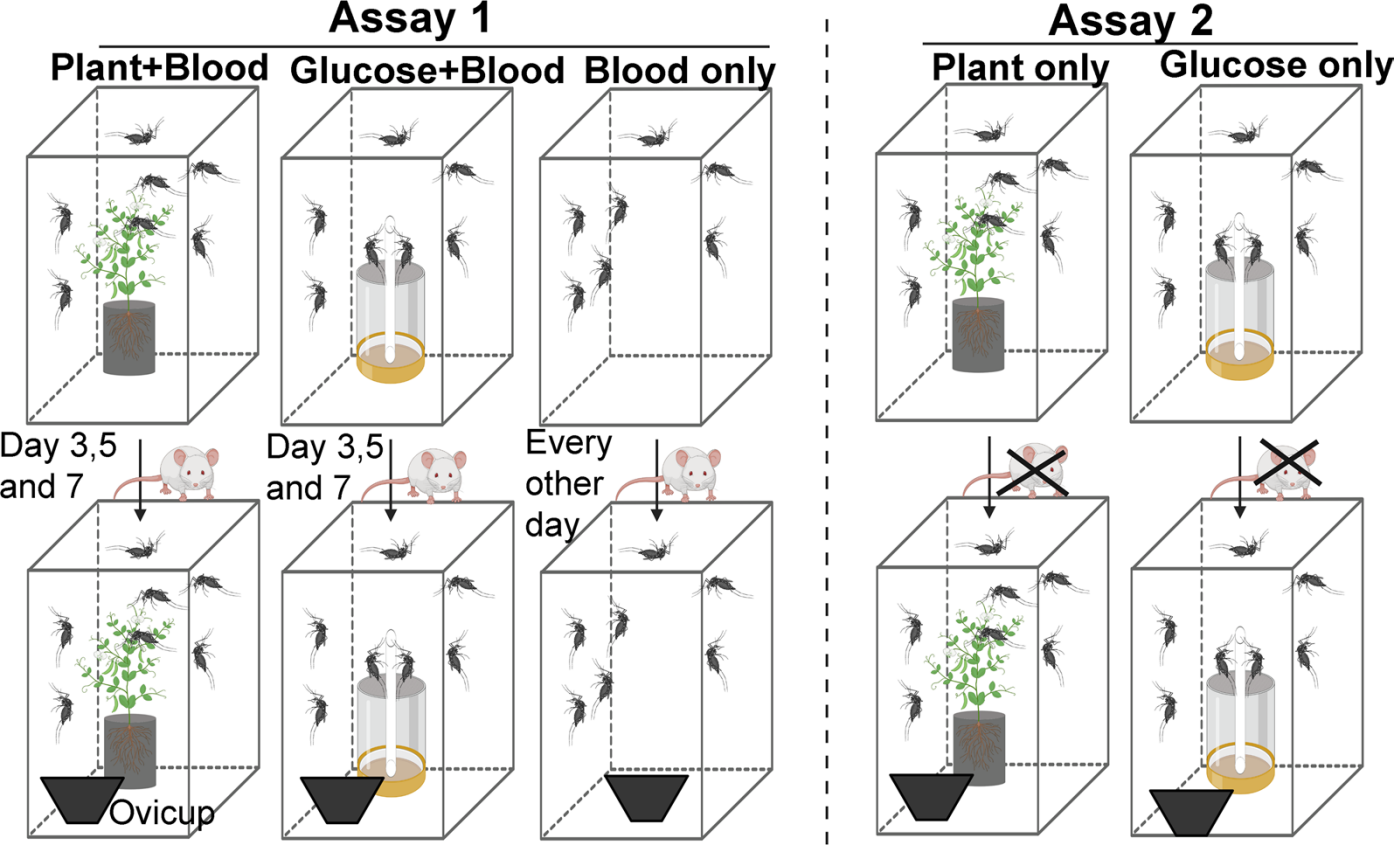
Schematic representation of the study design. Mosquitoes were either fed on mouse blood (Assay 1) or no blood at all (Assay 2). Ovicups were placed in the cages on day 5 and monitored daily for eggs over the 30-day assay period; eggs were collected and the cups replaced with fresh ones every day. Sugar comprised 6% glucose solution. The images were created with BioRender.com

In Experiment I carried out in Kilifi, two assays were conducted. In the first assay, a group of 100 males and 100 females was introduced into a 30 × 30 × 100 cm cage containing *P. dulce* cuttings. In addition to the plant, which was continuously available, they were provided with initial three blood meals from mice at day 3, 5 and 7 from the onset of the assay (Fig. 1, Assay 1). The blood meal was provided by placing an anesthetized mouse on top of the mosquito cages and allowing the mosquitoes to feed on them for an hour. Oviposition cups were provided in all the cages 48 h after the first blood meal. They were monitored for survival and fecundity daily for 30 days. Mortality and the daily number of eggs laid were recorded and fresh oviposition cups provided. Control experiments comprised 100 females and 100 males of *Ae. aegypti* with access to (i) 6% glucose solution (the concentration routinely used in mosquitoes reared at *icipe*) plus three initial blood meals (Fig. 1, Assay 1) and (ii) blood meals only on alternating days for 30 days with a total of 15 blood meals. Nine living female mosquitoes were randomly selected from each replicate of all the treatments on day 15 for amino acid analysis as described below. The choice of day 15 for nutrient analysis was informed by the need to allow enough time for all blood-derived nutrients to be absorbed/cleared from the mosquito gut and increase detection of plant-derived nutrients. The second assay was the same as the one above, but no blood meal was provided (Fig. 1, Assay 2). Nine living female mosquitoes were randomly selected from each replicate of all the treatments on day 15 for amino acid and sugar analysis as described in Additional file 1. A total of three replicates using three different batches of mosquitoes were carried out for all nutrient regimes for both assays.

Similar experimental setup as above was used in Experiment II for survival and fecundity assays using *L. nepetifolia* and *O. ficus*-*indica*. Newly emerged females and males (100 mosquitoes for each sex) were provided with *L. nepetifolia*, *O. ficus*-*indica*, 6% glucose solution or mouse blood meal provided on alternate days. Except for the latter group, the mosquitoes in all the other three groups were either provided an initial three mouse blood meals on days 3, 5 and 7 (Fig. 1, Assay 1) or no blood meal at all (Fig. 1, Assay 2). Survival, fecundity and mosquito sampling for nutrient analysis was done as described above. The collected eggs were wrapped between moist paper towels and put in covered plastic trays for 48 h and then transferred to a dark container and allowed to dry slowly under rearing conditions [temperature of 28 (± 1)  °C and relative humidity of 80 (± 5)%].

To measure the hatchability of the laid eggs, the eggs were put in 18 × 12.5 × 2.1-inch trays and distilled water added to a depth of 1 inch. The water was first boiled and then allowed to cool to room temperature before introducing the eggs. The eggs were hatched according to the date laid and nutrient source. The number of larvae was counted daily for up to 2 weeks after which the unhatched eggs were considered not viable. The counted larvae were promptly removed.

### Analysis of host plant nutrient content and the ingested equivalence in *Aedes aegypti*

To understand the differences in the performance of *Ae. aegypti* on different nutrient sources, we quantified the amount of amino acids in the three plant species and the corresponding amounts ingested by the mosquitoes. Both plant sap from phloem in the succulent tissues and nectar from the nectaries were collected from the three plants using 20 µl micro-capillary tubes (Drummond Scientific Company, Brumall, PA, USA) tapered on one end using a glass puller. Between 10 and 30 µl of plant sap and nectar was separately collected, transferred into 1.5-ml low-binding Eppendorf tubes, snap frozen in liquid nitrogen and immediately stored at − 80 °C until analysis. Up to 100 µl of venous blood was drawn from mouse facial veins into 1.5-ml low-binding Eppendorf tubes and immediately snap-frozen in liquid nitrogen and stored at − 80 °C. Blood was collected from three mice drawn from different litters for both experiments conducted at KEMRI-Wellcome Trust Laboratories and *icipe*. The mosquito samples were prepared by dissecting mosquitoes preserved from survival assays and pooling the midgut plus crop (hereafter referred to as gut) from three mosquitoes.

To detect the amino acid content, the pooled guts, 10 µl of plant sap + nectar or 10 µl of blood samples was hydrolyzed with 6 M HCl for 3 h as described by Moran-Palacio et al. (2014). The product was re-suspended in 200 µl mmol 1-1 EDTA solution, pH 7.5 and incubated for 90 min in the dark in a sealed chamber equilibrated at 25 °C with a dish of saturated KH_2_PO_4_ to maintain high humidity. The EDTA samples were subsequently diluted in a water-acetonitrile solvent mixture in a ratio of 80:20 and analyzed on a liquid chromatograph attached to 6120 quadrupole mass spectrometer (Agilent Technologies). Samples were injected via the Agilent Technology 1260 Infinity series sample manager, injecting 10 µl on to the Agilent SB-C18 3.5 µm 4.6 × 250 mm column. The run time was 22 min at a flow rate of 0.7 ml/min. The solvent system consisted of A (water + 1% formic acid) and B (acetonitrile + 1% formic acid). The mobile phase used a gradient program, initially 95:5 (A: B), to 70:30 at 3 min, 20:80 at 7.5 min, 0:100 at 13 min and 95:5 at 20 min. The mass spectrometer was operated in positive ion mode, with a capillary voltage of 3 kV, with voltage 50–180 eV and mass range 50–300 m/z. The source temperature was 130 °C, desolvation temperature 350 °C, desolvation gas flow 100 ml/min (nitrogen) and con gas flow 0.7 ml/min (nitrogen). The amino acids were identified by comparing their mass spectra with the literature data [28].

The sugar contents of guts from males and non-blood fed female *Ae. aegypti* fed on different nutrients were analyzed as described in supplementary information (Additional file 1).

### Statistical analyses

For each nutrient regime, survival data from the three replicates was first subjected to GLM to determine if they could be pooled. Since no difference was detected, data from the three replicates were pooled and the difference in survival times of adult *Ae. aegypti* on different nutrient sources detected using Kaplan-Meier and Cox regression survival analyses. Mosquitoes sampled for nutrient analyses and those surviving after the 30-day observation period were treated as censored. The number of eggs laid on each day was corrected to the number of surviving females, and differences in fecundity between mosquitoes held on different nutrient sources were detected using zero-inflated GLM. The differences in hatching rate from different nutrient sources were compared using one-way ANOVA and Tukey *post-hoc* test. The gut amino acid content was quantified for the different nutrient sources and the differences detected using one-way analysis of variance. The differences in gut sugar content was detected using ANOVA and Tukey *post-hoc* test. All statistical analyses were done in R software version 3.6.3 [29].

## Results

### Host plants variably support *Ae. aegypti* survival, fecundity and egg viability

**Fig. 2:**
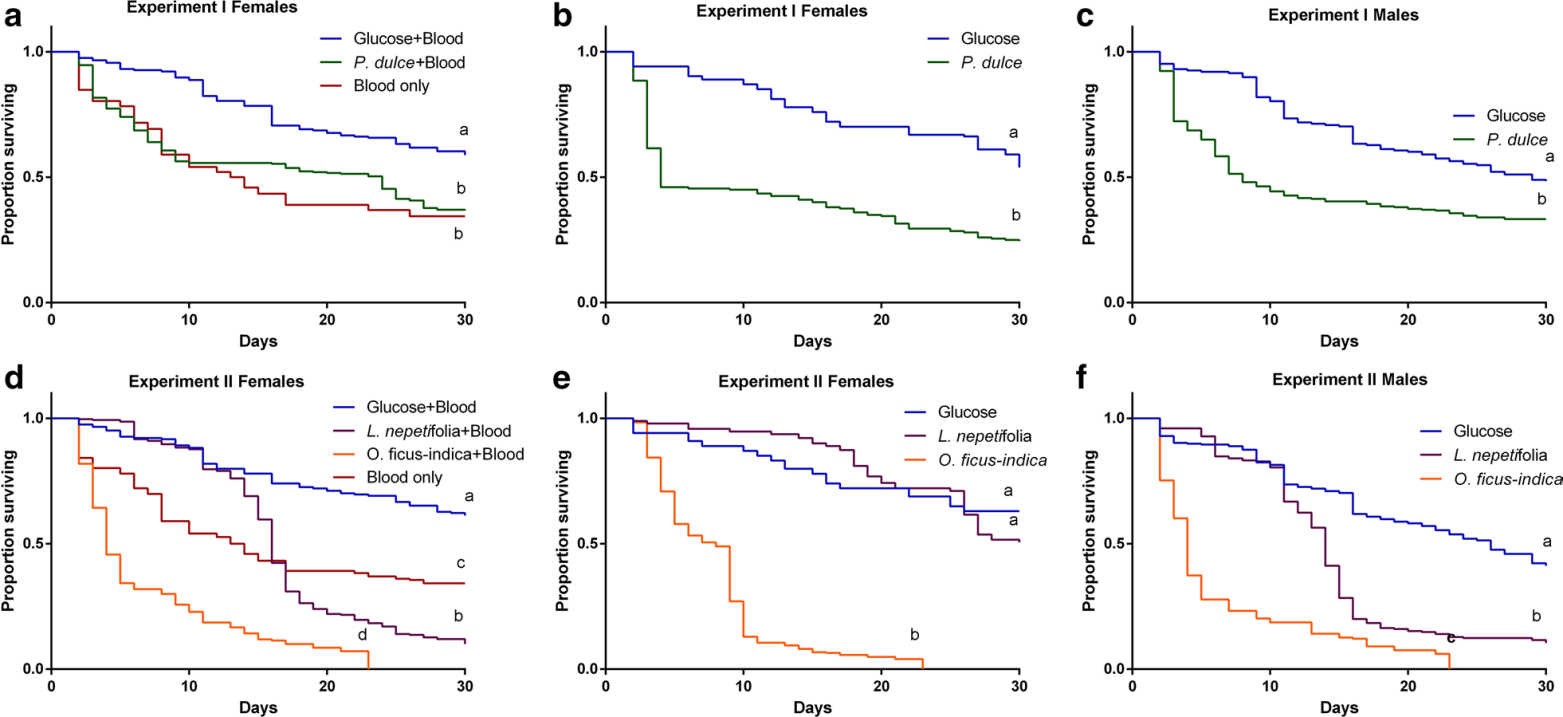
The impact of plant nutrients on *Ae. aegypti* male and female survival. **a** Survival curves of female *Ae. aegypti* on *P. dulce*, 6% glucose solution and exclusively mouse blood, with an initial three blood meal rations offered to those held on *P. dulce* and glucose solution (*P*-value < 0.001). (**b**–**c**) Survival curves of female and male *Ae.aegypti*, respectively, fed on *P. dulce* and glucose without initial blood meal (*P*-value < 0.001). **d** Survival curves of females held on *L. nepetifolia, O. ficus*-*indica,* 6% glucose solution and exclusively mouse blood, with the former three offered three initial blood meal rations (*P*-value < 0.001). (**e**–**f**) Survival curves of female and male *Ae. aegypti*, respectively, fed on *L. nepetifolia, O. ficus*-*indica* or glucose solution without an initial blood meal (*P*-value < 0.001). Survival curves denoted by different letters are significantly different. Differences in survival curves were detected using Kaplan-Meier analysis and Cox regression analyses

In Experiment I, *Ae. aegypti* fed on glucose survived significantly longer (23.5 days) than those fed on *P. dulce* and blood (17.7 days) or blood alone (16.1 days) (log rank = 40.785, df = 2, *P* < 0.001; Fig. 2a). With no initial blood meal, the mean survivals of female *Ae. aegypti* on glucose and *P. dulce* were 23.6 ± 0.8 and 13.1 ± 0.8 days, respectively (log rank = 48.04, df = 1, *P* < 0.001; Fig. 2b). Similar survival patterns were observed in males, with those fed on glucose having a mean survival of 21.6 ± 0.7 days while those fed on *P. dulce* had a median survival of 14.6 ± 0.7 days (log rank = 25.162, df = 1, *P* < 0.001; Fig. 2c).

In Experiment II, survival of *Ae. aegypti* females provided with an initial blood meal and fed on the different diets was significantly different (log rank = 419.727, df = 3, *P* value < 0.001; Fig. 2d), with mean survival of 24.0 ± 0.6, 17.0 ± 0.4, 7.2 ± 0.4 and 16.1 ± 0.8 days among glucose, *L. nepetifolia, O. ficus*-*indica* and blood, respectively. With no initial blood meal, the mean survivals on glucose solution, *L. nepetifolia* and *O. ficus*-*indica* were 23.9 ± 0.7, 25.2 ± 0.5 and 7.4 ± 0.2 days for females (log rank = 485.405, df = 2, *P*-value < 0.001; Fig. 2e) and 21.0 ± 0.6, 14.5 ± 0.5 and 6.5 ± 0.4 days for males (log rank = 354.639, df = 2, *P*-value < 0.001; Fig. 2f), respectively.

**Fig. 3:**
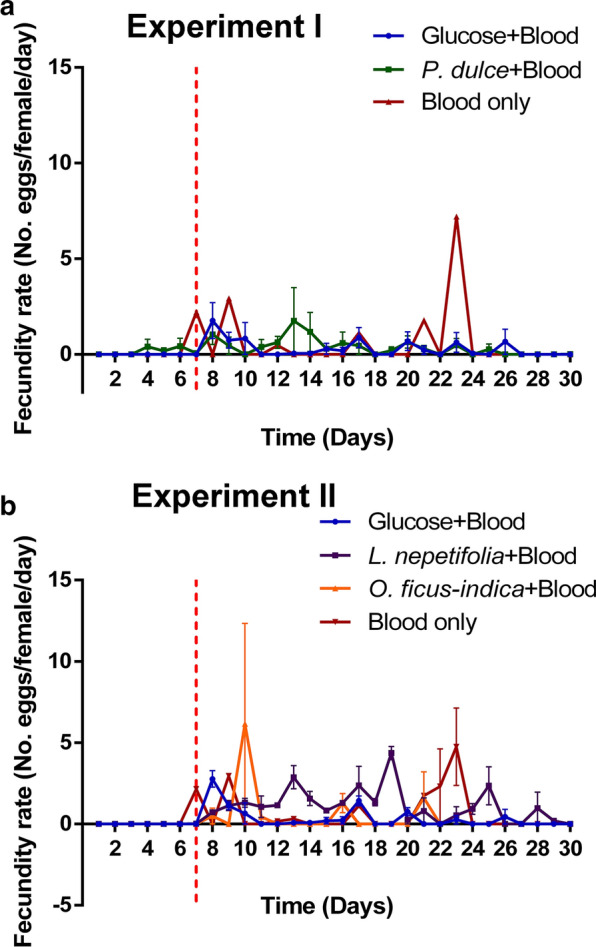
Effect of plant feeding on the fecundity of *Ae. aegypti*. **a** Line plots showing the oviposition patterns of mosquitoes fed on *P. dulce*, glucose and exclusively mouse blood meals. **b** Line plots showing the oviposition patterns of mosquitoes fed on *L. nepetifolia, O. ficus*-*indica*, glucose and exclusively mouse blood meals. The broken red line shows the day when the third blood meal was provided

For fecundity, females fed on *P. dulce* and glucose laid 1.6- and 2.2-fold fewer eggs, respectively, than those fed exclusively on blood but no significant difference was detected (F_(2, 267_) = 1.985, I = 0.139). On the other hand, those fed on *L. nepetifolia* laid 1.6-fold more eggs than those fed exclusively on blood, while mosquitoes fed on *O. ficus*-*indica* and glucose had 1.7- and twofold fewer eggs than those fed exclusively on blood meals, respectively (F_(3, 356_) = 3.495, *P *= 0.0158). Besides having the highest fecundity rate, mosquitoes fed on *L. nepetifolia* had a sustained moderate oviposition throughout the experimental period which was comparable to those that exclusively fed on blood meals (Fig. 3a, b).

**Fig. 4:**
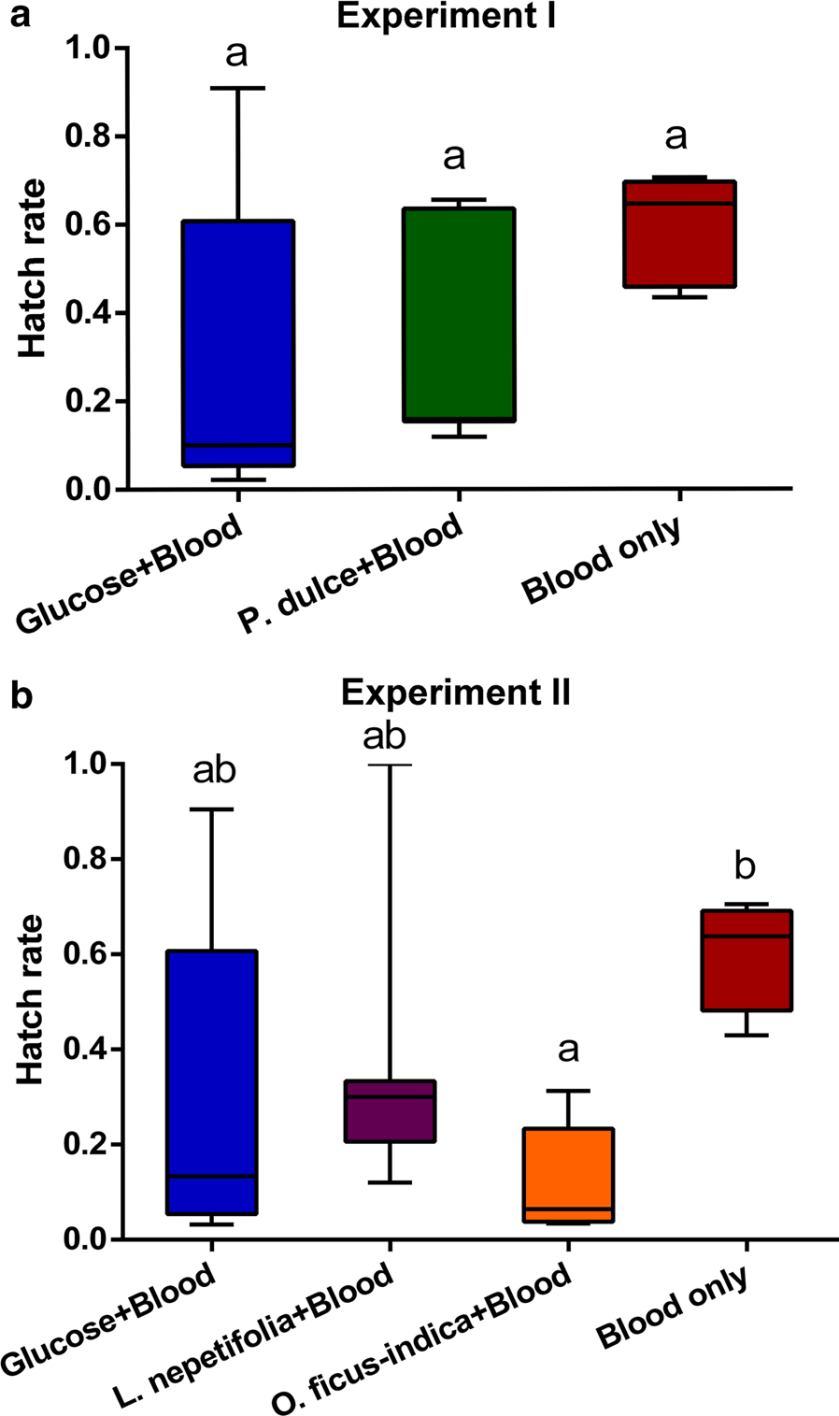
Effect of plant feeding on the viability of *Ae. aegypti* eggs. **a** Hatching rates of eggs laid by mosquitoes fed on 6% glucose, *P. dulce* and exclusively blood meals. The difference in hatching rates was detected using ANOVA and Tukey *post-hoc* test (*P*-value = 0.0484). **b** Hatching rates of eggs laid by mosquitoes fed on 6% glucose, *L. nepetifolia, O. ficus*-*indica* and exclusively blood meals. Box plots denoted by different letters are significantly different. The difference in hatching rates was detected using one-way ANOVA and Tukey *post-hoc* test

Regarding egg viability, marginally significant differences were detected in the median hatching rates of eggs laid by mosquitoes fed on glucose (0.10), *P. dulce* (0.0.16) and blood (0.65) (F_(2, 31_) = 3.344, *P *= 0.0484; Fig. 4a). On the other, a significant difference was detected in the hatching rates of eggs from Experiment II (F_(3,40_) = 4.221, *p *= 0.011), with median hatching rates of 0.13, 0.30, 0.06 and 0.64 for eggs from females fed on glucose, *L. nepetifolia, O. ficus*-*indica* and blood meal, respectively (Fig. 4b).

### Variable nutrient quality support: observed differences in the fitness matrix of *Ae. aegypti* fed on different host plants

**Fig. 5:**
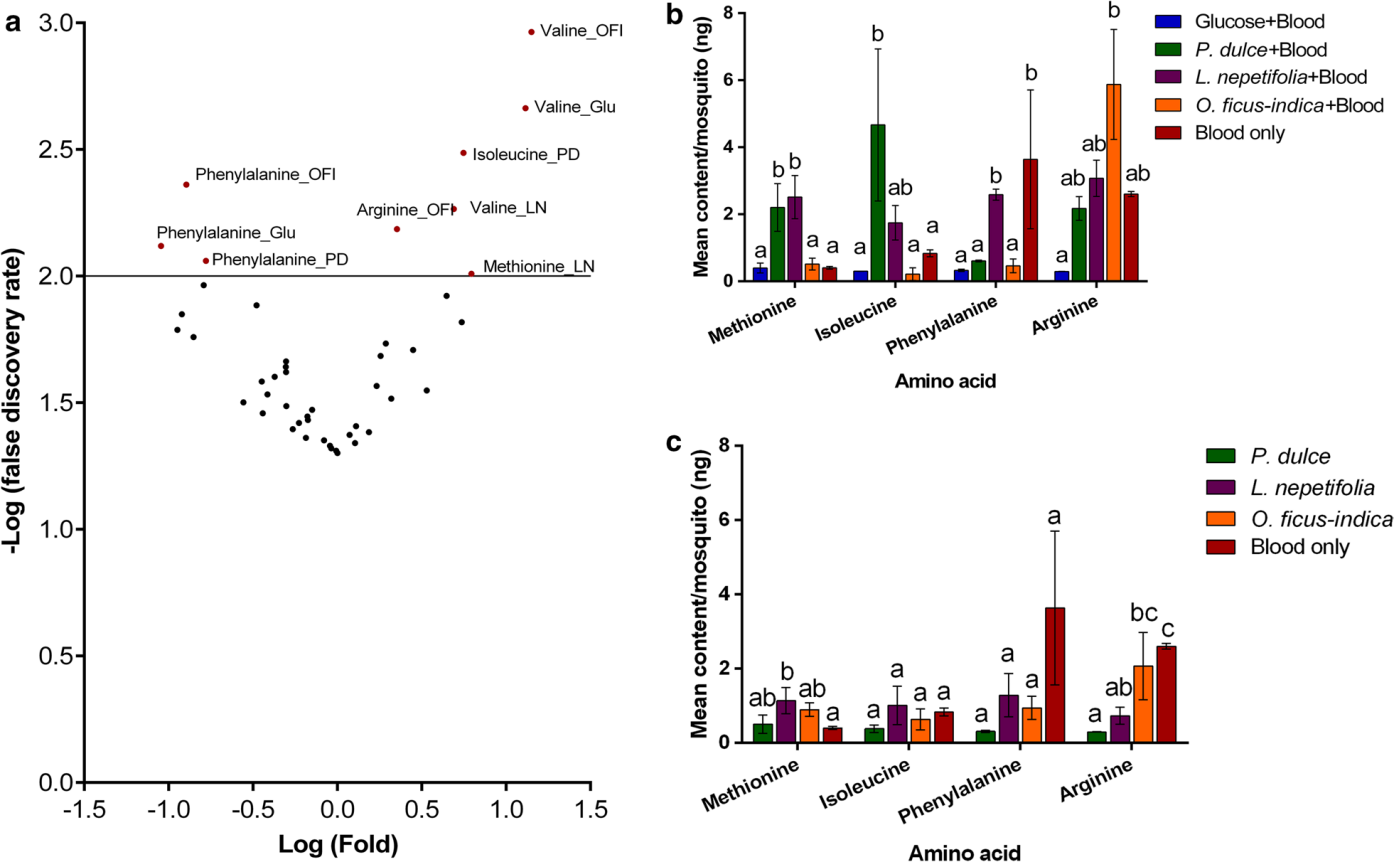
Amino acid content of gut of *Ae. aegypti* fed of different nutrient sources. **a** Volcano plot depicting unique amino acids significantly abundant or low in the guts of females fed on *P. dulce, L. nepetifolia, O. ficus*-*indica* and 6% glucose solution compared to those exclusively fed on mouse blood. The horizontal line shows where *P* = 0.05 by F statistics. The significantly high or low abundant amino acids are shown. *LN* denotes *L. nepetifolia, PD* denotes *P. dulce*, *OFI* denotes *O. ficus*-*indica*, *Glu* denotes glucose solution. **b** Mean amounts of the unique amino acids in the guts of females fed on the five nutrient sources with three initial blood meals. The *P*-values were < 0.001, < 0.01, < 0.05 and < 0.01 for methionine, isoleucine, phenylalanine and arginine, respectively. **c** Mean amounts of the unique amino acids in the guts of females fed on the three plant nutrients with no initial blood meals and those fed exclusively on blood. The *P*-values were < 0.05, = 0.25, = 0.052 and < 0.01 for methionine, isoleucine, phenylalanine and arginine, respectively. The differences in gut amino acid content were detected by one-way ANOVA and Tukey *post-hoc* test. Bars denoted by different letters are significantly different

A total of 12 amino acids present in mouse blood were detected in variable amounts in the sap plus nectar of three plant species. These included valine, serine, glutamine, proline, glycine, methionine, tyrosine, isoleucine, leucine, phenylalanine, tryptophan and arginine. Uniquely abundant amino acids detected in the guts of mosquitoes fed on the nutrient regimes included valine, arginine, isoleucine, methionine and phenylalanine (Fig. 5a). Valine was 5-, 13- and 14-fold more abundant in the guts of mosquitoes fed on *L. nepetifolia,* glucose and *O. ficus*-*indica*, respectively. In addition, arginine was abundant in the guts of those fed on *O. ficus*-*indica*, isoleucine in those fed on *P. dulce* and methionine in those fed on *L. nepetifolia* (Fig. 5b). On the other hand, phenylalanine was 6-, 8- and 11-fold less abundant in the guts of mosquitoes fed on *P. dulce, O. ficus*-*indica* and glucose solution, respectively, relative to those exclusively fed on blood (Fig. 5b). Notably, glutamic acid was present in the guts of mosquitoes fed on mouse blood but absent in those from all the other diets. To further confirm that female *Ae. aegypti* indeed were able to imbibe these amino acids from their host plants, we analyzed four of the identified amino acids in the guts of non-blood-fed mosquitoes. Besides valine, none of the females from the glucose diet group had any detectable amino acids in their guts. However, females fed on all three host plants had variable amounts of methionine, isoleucine, phenylalanine and arginine in their guts (Fig. 5c).

No significant differences were detected in the amount of sugar ingested by male *Ae. aegypti* (F_(3,93_) = 1.389, *P *= 0.251; Additional file 1: Fig. S1). However, females fed on *L. nepetifolia* imbibed significantly more sugar compared to those fed on *O. ficus*-*indica* (F_(3,93_) = 2.791, *P *= 0.0449; Additional file 1: Fig. S1).

## Discussion

Our findings show that the three plants used in this study differentially impact the survival and reproductive fitness of dengue vector, *Ae. aegypti*. We previously reported a sugar feeding frequency of 17% in female *Ae. aegypti* collected around vegetations in the coastal area of Kenya [16]. The study by Olson et al. [17] demonstrated that sugar feeding occurs at a much higher frequency than previously reported, with collection method and season being important in influencing the proportion of fructose-positive females captured. Plant sugars, particularly fructose, have been shown to provide a ready source of energy for various metabolic processes in several mosquito species [17, 30–33]. Extended survival time is pivotal in the transmission of vector-borne diseases as it guarantees completion of extrinsic incubation of the causative agents and increases the chances of multiple infective vertebrate host bites [34]. These findings further reinforce the argument of the central role played by plants in the biology of *Ae. aegypti*, contrary to previous beliefs [19–22].

While no eggs were laid by mosquitoes exclusively fed on the three plant species, significant differences in fecundity were observed when mosquitoes fed on them with initial blood meal rations. Those fed *L. nepetifolia* had slightly higher oviposition than their exclusively blood-fed counterparts, while those fed on *P. dulce, O. ficus*-*indica* and glucose laid fewer eggs than blood-fed females. Similar impacts of plant diets on mosquito fecundity have been observed for *An. gambiae* [30, 35] and *Culex pipiens* [33]. Although sugar feeding has long been suggested to impact the fecundity of *Aedes* mosquitoes [36, 37], to the best of our knowledge, this is the first evidence directly linking plant feeding to *Ae. aegypti* fecundity. Plant nectars have been shown to increase mating competence in males of different mosquito species [31, 32, 38, 39]. Sugar has also been shown to be important in inducing egg development in autogenous *Ae. albopictus* and *Cx. pipiens f. molestus* [40, 41]. The failure of *Ae. aegypti* to lay eggs without an initial blood meal in this study is not surprising, although varying degrees of autogeny has been reported among these species in East Africa [42, 43]. However, the potential of *L. nepetifolia* to not only boost their overall fecundity but also induce a sustained oviposition long after the last blood meal is noteworthy. Although lower than the hatching rates in eggs from exclusively blood-fed females, a good proportion of eggs laid by females fed on *L. nepetifolia* were viable. The difference in fecundity of mosquitoes held on different nutrient sources observed in this study can be explained by three propositions: (i) males fed on *L. nepetifolia* had sugar-rich diets and therefore increased mating competence and reproductive output in females compared to males from the exclusively blood meal diet, which died off within 3 days; (ii) females held on *L. nepetifolia* imbibed sufficient sugar meals/sap from the succulent plant tissues, thereby resulting in constant distention of the abdomen and inducing oocyte maturation following the initial blood meal, as has been reported for *Ae. albopictus* [40]; (iii) mosquitoes feeding on the three plants, especially on *L. nepetifolia*, imbibed not only sugar but also amino acids, which supplemented those received from the initial blood meal, further boosting their reproduction.

We explored the third proposition further by analyzing the amino acid content of female *Ae. aegypti* held on these plant species and comparing the outputs with those from mosquitoes fed exclusively on blood and their plant sources. Twelve amino acids present in mouse blood were positively identified in the plant sap/nectar and the guts of blood-fed females held on different diets in varying proportions. Notably, mosquitoes held on *L. nepetifolia* had high methionine content in their guts, while those held on *P. dulce* had high isoleucine content. Intriguingly, phenylalanine was significantly low in females held on *P. dulce*, *O. ficus*-*indica* and glucose solution, with mosquitoes fed on the three plant species and glucose lacking glutamic acid. Non-blood-fed females held on the three plant species had similar amino acid profiles as those offered an initial blood meal. These observations support our proposition that female *Ae. aegypti* imbibed variable amounts of amino acids from these plant species, which differentially impacted their fecundity. This was further supported by the detection of these amino acids in the respective host plants on which the mosquitoes were held. Different amino acids have been shown to impact differently on mosquito fecundity. Phenylalanine and tyrosine have been shown to be important for the development and tanning of *An. gambiae* and *Ae. aegypti* eggs [44, 45], while isoleucine is important in follicular maturation and preventing egg resorption in *Ae. aegypti* [46]. Methionine and leucine have been shown to increase the fecundity of green pea aphid, *Cyrthosiphon pisum* by enhancing the target of rapamycin (TOR) signaling pathway [47]. This study represents the first empirical evidence of the possible involvement of plant-derived amino acids in the reproductive fitness of *Ae. aegypti*.

We acknowledge that the study has some limitations that warrant further consideration: (i) in this study, individual females that blood fed were not separated from those that did not blood feed; (ii) we did not include a control with sugar continuously available and a blood meal provided every other day to allow males to survive longer and improve their mating competence; (iii) the use of plant cuttings in the assays with *P. dulce* and whole potted plants in the case of *L. nepetifolia* and *O. ficus*-*indica* could have contributed to the observed differences in the performance of *Ae. aegypti*. Although *L. nepetifolia* significantly improved the survival and fecundity of *Ae. aegypti*, it was not identified as a natural host plant of this species in our previous study and is not found within the locality where their eggs were collected. However, it serves as an important pointer to the potential of other plants with similar nutritional value to impact mosquito vector populations.

## Conclusion

We conclude that these findings offer significant insight into the role of plant-derived nutrients in the biology and population dynamics of *Ae. aegypti*. Our study provides empirical evidence that plant-derived amino acids such as phenylalanine, methionine, isoleucine and arginine could play a role in sustaining the reproductive fitness of these disease vectors in situations where vertebrate hosts are scarce and only a few blood meal encounters are possible.

## Supplementary information

**Additional file 1.** Methods and results of *Aedes aegypti* gut sugar content analysis.

## Data Availability

The datasets used and/or analysed during the current study are available from the corresponding author on reasonable request.
