# Supplementary material for: Plant nutrient quality impacts survival and reproductive fitness of the dengue vector Aedes aegypti
Source: Parasit Vectors. 2021 Jan 4;14:4. doi: 10.1186/s13071-020-04519-y (PMC7783993; doi:10.1186/s13071-020-04519-y)
Supplement: Supplementary file 1 — Additional file 1. Methods and results of Aedes aegypti gut sugar content analysis. [file 13071_2020_4519_MOESM1_ESM.docx]

**Additional file 1**

**Gut sugar content analysis**

For sugar analysis, the pooled gut + crop samples were transferred to a 5 ml reaction vial, 100 µl of pyridine added and allowed to macerate for three days. The samples were then derivatized by adding 100 μl N-Methyl-bis trifluoro acetamide (MBTFA) (Sigma, 98%) and incubating them at 60 °C for 1 hr. The products were sieved through glass wool and subjected to GC/MS analysis. GC/MS analysis was carried out in the splitless injection mode using an Agilent Technologies 7890 gas chromatograph coupled to a 5975C inert XL EI/CI mass spectrometer (EI, 70 eV, Agilent, Palo Alto, CA) equipped with an HP-5 column (30 m × 0.25 mm ID × 0.25 μm film thickness, Agilent, Palo Alto, CA). Helium was used as the carrier gas at a flow rate of 1.2 ml/min. The oven temperature was held at 35 °C for 3 min, then programmed to increase at 10°C/min to 280 °C and maintained at this temperature for 10 min. The ions 69 and/or 319 were initially used to identify plant sugars from the resulting chromatograms. Plant sugars were then identified by comparison of spectra of their trimethylsilyl derivatives with library data (NIST05aL.Software), and with those of authentic standards derivatized similarly. Three biological replicates with two technical replicates each was obtained for each nutrient regime. Similarly, equal amounts of plant sap and nectar from each plant species were separately mixed and 10 µl of the resulting mixture subjected to derivatization reaction and sugar analysis as described above. For quantification, a calibration curve was prepared by derivatization and analysis of 0.01, 0.1, 1, 10 and 100 μg of synthetic glucose, fructose, rhamnose, mannose, sucrose and maltose and analyzing them on GC/MS as described above. The absolute peak areas were used to generate external calibration curves.





Figure S1. Gut sugar content of Ae. aegypti fed on different nutrient sources. Total sugar content of the eight identified sugars in the gut of male and non-blood fed female Ae. Aegypti were identified and quantified by GC/MS. The difference in sugar content between the different nutrient sources was detected using one-way ANOVA and Tukey Post Hoc test (p = 0.251 and 0.0449 for males and females, respectively).
